# Dietary Patterns and Breast Cancer Risk: A Multi-Centre Case Control Study among North Indian Women

**DOI:** 10.3390/ijerph15091946

**Published:** 2018-09-06

**Authors:** Krithiga Shridhar, Gurpreet Singh, Subhojit Dey, Sarvdeep Singh Dhatt, Jatinder Paul Singh Gill, Michael Goodman, Melina Samar Magsumbol, Neil Pearce, Sandeep Singh, Archna Singh, Preeti Singh, Jarnail Singh Thakur, Preet Kaur Dhillon

**Affiliations:** 1 Centre for Chronic Disease Control, Centre for Chronic Conditions and Injures, Public Health Foundation of India, Gurgaon 122002, India; g.krithiga@phfi.org; 2 Postgraduate Institute of Medical Sciences and Research, Chandigarh 160012, India; gsinghpgi@gmail.com (G.S.); sdhatt@yahoo.com (S.S.D.); jsthakur64@gmail.com (J.S.T.); 3 Indian Institute of Public Health, Public Health Foundation of India, Gurgaon 122002, India; subhojit_dey@hotmail.com (S.D.); arch_singh@ymail.com (A.S.); 4 Guru Angad Dev Veterinay and Animal Sciences University, Ludhiana 141004, India; gilljps@gmail.com; 5 Rollins School of Public Health, Emory University, Claudia Nance Rollins Building, 1518 Clifton Road, CNR 7040H, Atlanta, GA 30322, USA; mgoodm2@emory.edu; 6 Public Health Foundation of India, Gurgaon 122002, India; melina@phfi.org (M.S.M.); preeti.kbains@gmail.com (P.S.); 7 London School of Hygiene and Tropical Medicine, London WC1E 7HT, UK; neil.pearce@lshtm.ac.uk; 8 Centre for Human Genetics and Molecular Medicine, Central University of Punjab, Bathinda 151001, India; sandeepsingh82@gmail.com; 9 All India Institute of Medical Sciences, New Delhi 110029, India; 10 Indian School of Business, Sahibzada Ajit Singh Nagar, Punjab 160062, India

**Keywords:** breast cancer, diet, vegetarian, egg, India

## Abstract

Evidence from India, a country with unique and distinct food intake patterns often characterized by lifelong adherence, may offer important insight into the role of diet in breast cancer etiology. We evaluated the association between Indian dietary patterns and breast cancer risk in a multi-centre case-control study conducted in the North Indian states of Punjab and Haryana. Eligible cases were women 30–69 years of age, with newly diagnosed, biopsy-confirmed breast cancer recruited from hospitals or population-based cancer registries. Controls (hospital- or population-based) were frequency matched to the cases on age and region (Punjab or Haryana). Information about diet, lifestyle, reproductive and socio-demographic factors was collected using a structured interviewer-administered questionnaire. All participants were characterized as non-vegetarians, lacto-vegetarians (those who consumed no animal products except dairy) or lacto-ovo-vegetarians (persons whose diet also included eggs). The study population included 400 breast cancer cases and 354 controls. Most (62%) were lacto-ovo-vegetarians. Breast cancer risk was lower in lacto-ovo-vegetarians compared to both non-vegetarians and lacto-vegetarians with odds ratios (95% confidence intervals) of 0.6 (0.3–0.9) and 0.4 (0.3–0.7), respectively. The unexpected difference between lacto-ovo-vegetarian and lacto-vegetarian dietary patterns could be due to egg-consumption patterns which requires confirmation and further investigation.

## 1. Introduction

Breast cancer is the leading malignancy in terms of both site-specific incidence (43.1/100,000 person-years) and mortality (12.9/100,000 person-years) among women worldwide [1]. While the strongest risk factors for breast cancer are non-modifiable (e.g., family and reproductive history), other factors such as alcohol, smoking, physical activity, body composition and dietary habits can be modified and may offer useful targets for preventive measures [2]. The available evidence suggests that high intake of non-starchy vegetables, dairy products and diets high in calcium and antioxidant/anti-inflammatory nutrients may decrease risk for breast cancer in both pre- and postmenopausal women [2]. In addition, certain food components such as eggs have shown a non-linear ‘J-shape’ association with breast cancer risk [3] and eating fish rather than meat appears to lower the risk of breast cancer among postmenopausal women [4]. Overall, evidence suggests that while a healthy dietary pattern including fruits, vegetables, whole grains, lean protein and legumes may be protective, diets high in saturated fats, red and processed meats, added sugars, fried foods and refined grains may act as risk factors for breast cancer [5].

In India, breast cancer is the leading cancer site in most states. Although the average national incidence rate is lower than that in the West (Age-standardized rate/100,000 women: 21.6 vs. 90–110), [6] some regional rates (e.g., New Delhi and Punjab) are as high as the global rate at 33.9–41/100,000 women [7,8]. The disease characteristics in Indian breast cancer patients may be different from those observed in the US or Western Europe, with higher proportions of women diagnosed at a younger age and with a higher proportion of triple negative, high-grade and/or inflammatory tumors [9,10,11]. Indian dietary patterns—a blend of healthy and unhealthy components—are also distinct with strong cultural influences, often lifelong adherence and multi-generational [12,13], commonly used anti-carcinogenic spices such as turmeric [13], low levels of meat consumption (median intake: 20.3 (9.6–38.9) g/day [14,15] with <2% consuming meat daily [12]) and a high proportion of vegetarians [4,15,16,17,18]. Relatively few studies have investigated the association between major dietary patterns and breast cancer risk both in India [19,20,21,22] and among Indian migrants in the West [23,24]. A recent, large multi-centre hospital-based case-control study reported no difference in breast cancer risk among vegetarians and non-vegetarians, though there was no information on different types of vegetarian diet (lacto- versus lacto-ovo-vegetarian) and macro- or micro-nutrients to understand differences between the comparison groups [25]. Here, we report the findings from a multi-centre case control study of breast cancer that compares non-vegetarians to women who adhere to two common Northern Indian dietary patterns [lacto-vegetarianism (no animal product except dairy) and lacto-ovo-vegetarianism (no animal product except dairy and eggs)].

## 2. Methods

### 2.1. Study Participant Identification and Recruitment

This is a multi-centre case-control study conducted in 2013–2015 in the neighboring states of Punjab and Haryana in North India. The eligibility criteria for all female participants included age 30–69 years (at diagnosis for cases and the same date for matched controls) and residence in the study area for at least 10 years. Women with any prior treatment for breast cancer or history of another primary cancer were excluded from the study.

The study recruitment protocol included a hospital-based and population-based component. Cases were newly diagnosed, biopsy-confirmed breast cancer (International Classification of Disease-Oncology code C50) patients with a date of diagnosis from January 2013 through January 2015. Hospital-based cases were recruited from two large hospitals located in the study area (Post Graduate Institute of Medical Education and Research, Chandigarh and Acharya Tulsi Regional Cancer Centre, Bikaner) that serve the majority of cancer patients in the region. Population-based cases were recruited from the cancer registries. Cases had a date of diagnosis less than 6 months from the date of registration with any stage of disease, and could not have started treatment before recruitment into the study. Two population-based cases could not participate due to severe illness.

All controls were frequency matched to cases in 5-year age groups (30–34, 35–39 … 65–69 years) and state/territory of residence (Punjab or Haryana). Hospital-based controls were recruited among women receiving outpatient Orthopaedic care and residing within 25 km of the district headquarters of the cases and also matched to cases on date of diagnosis (within 6 weeks). Population-based controls were selected from a list of residents of the study areas supplied by population survey registers available at the village level, where for every recruited case, ten eligible controls were randomly sampled for participation in the study. These were women living in the same state and residing within 25 km of their district headquarters of the cases and also matched to cases by the type of location (urban, semi-urban and rural). Among population-based controls, 19 were unavailable at their home when visited, and 2 refused to participate.

We recruited eligible participants (*N* = 765; 404 cases and 361 controls) across the participating institutes and general population, of whom 11 had incomplete questionnaires (7 controls and 4 cases declined to respond in full due to time constraints) yielding a final sample size of 754 (400 cases and 354 controls). We enrolled 308 hospital-based cases and 256 hospital-based controls and 92 population-based cases and 98 population-based controls. The response rate exceeded 99% for both cases and controls with respect to population and hospital settings.

### 2.2. Dietary Exposure Assessment

Type of dietary pattern (lacto-vegetarian, lacto-ovo-vegetarian and non-vegetarian) and its duration in years were self-reported by the participants. Lacto-vegetarians were those who ate no eggs, meat of any type (including poultry) and fish; lacto-ovo-vegetarians were those who included eggs in their diet but no meat of any type and fish and non-vegetarians were those who ate eggs, meat of any type and/or fish. More than half (54%) of participants reported lifelong adherence to their dietary pattern; adherence for over 10 years and up to 10 years was reported in 24% and 10.5% of interviews, respectively; and the information on duration of adherence was missing for 11.5%.

Additionally, a sub-set of participants (*N* = 298; 170 cases and 128 controls) was asked to respond to a semi-quantitative, validated, 184-item food frequency questionnaire (FFQ) adapted from the Indian Migration Study [26]. The FFQ collected information on portion size and frequency of 184 commonly consumed food items over the last one year. Standard portion size (e.g., tablespoon, ladle, and bowl) and intake frequency (daily, weekly, monthly or yearly/never) were recorded after showing examples of vessels and different portion sizes. A Nutrient database, developed by the Aravind Eye Hospital, Madurai, India, [27,28] was used to calculate the macro and micro-nutrient content of each recipe using Indian food composition tables. The United States Department of Agriculture nutrient database (USDA, Release No. 14) or McCance and Widdowson’s Composition of Foods were used when nutrient values were unavailable from the Indian food composition tables.

### 2.3. Covariate Data Collection

Structured interviewer-administered questionnaire data and anthropometric measurements were collected on potential risk factors that may act as confounders and effect modifiers. Data on demographic and socioeconomic characteristics included age, residence, marital status, religion, education and occupation. Additional information included family history of breast cancer and other malignancies, and reproductive history including ages at menarche, first pregnancy and menopause, total number of pregnancies and children, breast feeding duration, and use of oral contraceptive pills. Women were classified as postmenopausal if they reported having undergone a natural menopause, bilateral oophorectomy or hysterectomy. Information on types and amounts of physical activity during occupational, recreational, household and commuting time periods was collected using the previously validated General Physical Activity Questionnaire [29]. The questionnaire-derived information was used to calculate frequency, duration (in minutes per week) and intensity (light, moderate and vigorous) of physical activity at work, at home, during travel and at leisure.

Weight was measured to the nearest 0.1 kg with a digital balance (SECA 899), and standing height was measured to the nearest 1 mm with a plastic stadiometer (Leicester height measure; supplied by Chasmors, London, UK). Measurements were taken twice and the average of two values was used in the analysis. Each participant’s weight and height were used to calculate body mass index (BMI), expressed as kg/m^2^. Waist circumference was measured using a non-stretch measuring tape with a narrow blade. Diabetes and hypertensive status were self-reported based on a doctor’s diagnosis.

### 2.4. Statistical Analysis

All data were presented as means (±standard deviations [SD]) or medians (interquartile ranges [IQR]) for continuous variables or as numbers (%) for categorical variables. Bivariate comparisons of cases and controls with respect to socio-demographic, lifestyle-related (including dietary), reproductive and anthropometric factors were carried out using appropriate tests for statistical significance depending on the type and distribution of variables under investigation.

We used multivariable unconditional logistic regression analysis adjusted for a priori selected confounders and matching variables. The covariates in fully adjusted models included age (years), region of residence (Punjab or Haryana), mode of recruitment (hospital- or population-based), education (college education, school education or no formal education), religion (Sikhism, Hinduism or others) family history of cancer (yes/no), physical activity (minutes/week), BMI (kg/m^2^), waist circumference (cm), duration of estrogen exposure (years), age at first pregnancy (years), breastfeeding per child (months) and history of diabetes (yes/no) and hypertension (yes/no). Total estrogen exposure in years was calculated as the difference between ages at menopause and menarche for post-menopausal women or the difference between age at recruitment and age at menarche for pre- or peri-menopausal women. Effect modification by menopausal status was tested by likelihood ratio tests for interaction of dietary patterns with menopausal status.

The same multivariable analysis of the association of dietary patterns and breast cancer risk was conducted separately in the subset of participants with FFQ data. Additional analyses were carried out to compare FFQ-derived nutrient intakes across dietary patterns. The nutrient intake levels for each category were expressed as medians (IQRs) and were accompanied by a Kruskal-Wallis test.

### 2.5. Ethics

All study participants provided informed consent. Information sheets in local language were given to the participants, and their signatures were obtained in the consent forms. Ethics committee approval was obtained from all participating institutions, which included the coordinating centre PHFI India (TRC IEC-170/13), as well as from study hospitals (P-371/PGI/IEC/2013/1690 and ECR/27/SP/2013) and from the population-based cancer registry, and the procedures followed were in accordance with the ethical standards of the committee.

## 3. Results

The study population consisted of 400 breast cancer cases and 354 controls (Table 1). The mean age of participants at recruitment into the study was 48.9 (±9.8) years, and the majority (87.4%) were housewives. Formal school and college-level education was reported in 44% and 14% of participants, respectively. Sikh (56.2%) and Hindu (34.1%) religions accounted for 90% of the population, 97.3% were ever married and 59.1% were post-menopausal. Tobacco consumption in any form was negligible in this population (<1%).

Despite matching, cases were on average 2 years older than controls at recruitment and had higher proportions of individuals with formal education (65.4% vs. 49.2% in controls; *p* < 0.001). Further, a higher proportion of the cases reported having diabetes (13.5% versus 5.9%; *p* = 0.001) and a family history of cancer (20.5% versus 13.3%; *p* = 0.009). Cases also had greater waist circumference (mean: 95.9 (14.8) cm versus 93.0 (13.5) cm; *p* = 0.0051) compared to controls, but the two groups did not differ with respect to BMI. Reproductive factors such as average age at first pregnancy (22.9 (3.7) years versus 21.9 (2.8); *p* < 0.0002) and number of years of estrogen exposure (29.4 (6.6) years versus 27.5 (6.7) years; *p* = 0.0357) were also higher among cases while physical activity (moderate to vigorous activity: 140 (0, 420) versus 240 (0, 750) min/week; *p* = 0.0003) was higher among controls.

Lacto-ovo-vegetarianism (62.1%) was the major dietary pattern reported in this population followed by lacto-vegetarianism (20.7%) and non-vegetarianism (17.2%). A majority reported following the dietary pattern either lifelong (53.9%) or over 10 years (24.0%) (Table 1). Missing information for any of the covariates constituted less than 5% of observations.

Overall, a higher proportion of non-vegetarians had a family history of cancer and reported having diabetes or hypertension; they also had greater waist circumference and BMI, higher age at first pregnancy and lower physical activity compared to lacto-ovo- and lacto-vegetarians (Supplementary Table S1). On the other hand, non-vegetarians were more likely to be school/college educated (74.5% vs. 54.5% among the rest; *p* < 0.001) and reported breastfeeding their children for a longer time (17.3 (±10.7) vs. 13.9 (±12.4) months per child; *p* = 0.0156).

Multivariate analyses revealed that lacto-ovo-vegetarians were less likely to have a diagnosis of breast cancer compared to either non-vegetarians (OR: 0.6; 95% CI: 0.3–0.9; *p* = 0.033) or lacto-vegetarians (OR: 0.4; 95% CI: 0.3–0.7; *p* < 0.001, Table 2) after adjusting for socio-demographic information, family and medical history, anthropometrics, lifestyle and reproductive risk factors as well as region of residence and mode of recruitment. The associations remained similar after excluding participants less than 30 years of age (*N* = 4) and were not modified by menopausal status.

All associations and levels of significance remained the same without accounting for BMI and waist circumference in fully-adjusted models.

When we evaluated the association in the subset of participants with FFQ data, the results were consistent with those observed in the full study population (Supplementary Table S2). While lacto-ovo-vegetarians did not differ significantly from lacto-vegetarians in terms of specific nutrients (Supplementary Table S3), a comparison of non-vegetarians to lacto-ovo-vegetarians demonstrated substantial differences. In particular, non-vegetarians were found to consume much higher quantities of cholesterol, choline, and several vitamins and vitamin precursors including beta-carotene, lutein/zeaxanthine, vitamin C and vitamin B12. The observed differences in nutrient intakes were similar when the data were restricted to controls with FFQ information (*N* = 128), but most corresponding comparisons were no longer statistically significant due to the small sample size (data not shown). When the same nutrient intakes were compared in cases and controls, a significant difference was only observed for cholesterol (Table 3).

## 4. Discussion

In this multi-centre case-control study conducted among North Indian women, we found an inverse association of lacto-ovo-vegetarian diet (diet with eggs but no fish or meat of any type) with breast cancer risk after adjusting for all important non-modifiable and modifiable risk factors. The inverse association was observed when compared to both non-vegetarian and lacto-vegetarian diet patterns. The associations were consistent in the sub-set of participants with FFQ estimated nutrient data. These associations did not differ by menopausal status both in the full study population and in the subset. The study findings did not support our hypothesis that a lacto-vegetarian diet has potential benefits against breast cancer risk in women, but a lacto-ovo-vegetarian diet was found to be inversely associated with breast cancer risk.

Evidence across different populations indicates an inverse association of breast cancer risk with healthy dietary pattern consisting of fruits, vegetables, legumes and whole grains and a positive association with a dietary pattern consisting of red and processed meat, fried foods and sugar [5]. The evidence on vegetarian diets is less convincing. In a meta-analysis of four cohort studies based in three Western countries (US, UK and the Netherlands), neither lacto-ovo-vegetarian diet nor pesco-vegetarian diet (fish-eating pattern) was associated with a discernable reduction in breast cancer risk [30].

An important distinguishing feature of traditional vegetarian diets in India is its prevalence (30% of women and 22% of men aged 15–49 years) [12] and heterogeneity for evaluating the different patterns of vegetarianism with sufficient power. In addition, dietary exposures are less likely to be affected by confounding due to other lifestyle factors such as tobacco [14,15,31]. Indian diets are influenced by cultural background and religious beliefs with lifelong patterns of adherence, and often multi-generational within families and communities [4,14,15,31]. Lacto-vegetarianism and non-vegetarianism followed by pesco- and lacto-ovo-vegetarianism are the major dietary patterns in India [31].

In our study population, over 60% were lacto-ovo-vegetarians followed by lacto-vegetarians (20%) and non-vegetarians (17%). A majority of participants (78%) followed their dietary pattern for more than 10 years and more than half reported lifelong adherence. This is aligned with nationwide surveys such as the National Family and Health Survey-5 (NFHS-5, 2015–16) for the states of Punjab and Haryana, where 75–79% of women do not consume fish or meat (including poultry) in their lifetime [12]. Our study is consistent with a study conducted in the southern Karnataka state, which observed a nearly three-fold higher risk for non-vegetarians compared to vegetarians [21], though there is no information on the prevalence of lacto-ovo-vegetarians. In contrast, a recent large multi-centre case control study from eight centers around India (*N* = 2101 cases and 2255 controls) found no association of life-long vegetarianism with breast cancer risk; however only 3% (*N* = 31) of participants in that study were lacto-ovo-vegetarians, so the study was underpowered to evaluate this effect [25]. Other smaller studies conducted in India or among Indian migrants in the West reported either null results [19] or weak non-significant inverse associations [23] with breast cancer risk. In light of NFHS-5 data and these studies, it is possible that our study population in India is uniquely powered to evaluate the association of lacto-ovo-vegetarian diets on breast cancer risk because of the high prevalence of this form of vegetarianism.

The distinguishing factor of lacto-ovo-vegetarians versus lacto-vegetarians in our study could be the intake of eggs and egg-based foods. Previous studies addressing this issue observed non-linear associations, with highest breast cancer risk observed among persons who either consume no eggs at all or report at least daily egg consumption [3,32]. A meta-analysis found linear increase in risk with intake of 2–5 eggs/week relative to 1 egg/week [33] and by contrast, a recent meta-analysis found no evidence of dose-response based on either linear or non-linear model assumptions [3]. An important distinction between our study and other studies that focused on egg consumption is confounding by meat intake. It is important to point out that strict lifelong lacto-ovo-vegetarianism may be common in India, but is less prevalent in other parts of the world.

The major dietary nutrients linking high intake of eggs with increased breast cancer risk include cholesterol, choline and protein intake relative to energy [3]. Cholesterol, a precursor of steroid hormones [33], is suggested to act through oestrogen receptors promoting cell proliferation [34] that is modified by pre-existing plasma levels of cholesterol and the state of diabetes [3]. Choline and high protein promote tissue growth and tumour progression, although choline has also been found to be inversely associated with breast cancer risk [3]. However, these biological mechanisms are not mechanistically proven and other factors such as cooking pattern, commercial cleaning of eggs with chlorine [3] and genetic susceptibility are speculated to play complex roles in the association of egg consumption with breast cancer risk [33]. Furthermore, eggs also contain certain amino acids, lutein, zeaxanthin, omega-3 and omega-6 fatty acids, which may be associated with reduced risk for breast cancer as observed in certain other studies [33].

In a sub-analysis of FFQ data, intakes of nutrients such as protein were slightly lower in lacto-ovo-vegetarian group compared to lacto-vegetarians, with the highest daily intakes of cholesterol and choline evident among non-vegetarian study participants. On the other hand, intakes of several presumably beneficial antioxidant/anti-inflammatory nutrients were higher in non-vegetarian than in both vegetarian groups. These observations may have several interpretations. First, it is plausible that the FFQ data did not capture important differences in nutrients such as fatty acids, or other more relevant pro- and anti-inflammatory components of the diet. Second, as reviewed elsewhere, [35] a reductionist approach to describing dietary patterns may be unable to take into consideration multilevel interactions and correlations among individual nutrients as well as interactions between various dietary and non-dietary factors. Finally, it is always possible that the lower breast cancer risk observed among participants who reported lacto-ovo-vegetarian diet might be a chance finding or a result of unaccounted systematic error attributable to imperfections of a case-control study design. However, when nutrient intakes were compared between cases and controls (78.1% of controls (*N* = 100/128) were lacto-ovo-vegetarians), a significant difference was only observed for cholesterol, suggesting modest egg consumption in our study population. Although we did not have this information in our study, the median egg consumption in Indian lacto-ovo-vegetarians (*N* = 205) across four different geographical regions (including participants from 18 states of India) was about 6 gm per day (3.1–13.1 gm) according to Indian Migration Study [14,15] with over 95% consuming <1 egg daily (unpublished data).

The nutrient values in our study are under-estimated compared to routine estimates in Indian populations [14,15]; however, it is unlikely that this underestimation differed across study groups. Thus, the FFQ data may still offer useful comparisons even if the absolute values for nutrient intake are lower than expected. While information on confounders in our study was extensive, we did not collect data on alcohol intake, a known risk factor for breast cancer in Western populations [2]. It is expected that alcohol plays little role as the recent NFHS-5 survey shows negligible consumption of alcohol among women in the states of Punjab (0.1%) and Haryana (0.1%) [12], so the potential confounding effect would be small.

These concerns notwithstanding, to the best of our knowledge, this is the first multi-centre case-control study in India that included a subset of population based cases and controls (in addition to hospital based cases and controls) to investigate the association between traditional dietary patterns and breast cancer risk among North Indian women, with additional nutrient information from a standardized, validated FFQ on a sub-set of participants. Another important feature of this study is the relative stability of dietary patterns, which may partially alleviate concerns about the accuracy of self-reports. Although FFQ data were only available on the subset of the study population, the main associations did not differ in this subset and in the full study population. In addition, detailed information about known modifiable and non-modifiable risk factors for breast cancer enabled adjustments for multiple confounders. Future detailed investigations on egg consumption patterns, and nutrient intake, along with cooking methods in this population are highly recommended.

## 5. Conclusions

Our results suggest that lacto-ovo-vegetarian, but not lacto-vegetarian dietary patterns may be associated with lower risk of breast cancer among North Indian women. While this could be attributed to egg consumption patterns, the mechanisms of the observed association are not clear. The observed difference between lacto-ovo-vegetarian and lacto-vegetarian dietary patterns requires confirmation, and if confirmed, further investigation.

## Figures and Tables

**Table 1:** Characteristics of 754 study participants.

Participant Characteristics	Total (*N* = 754)	Controls (*N* = 354)	Cases (*N* = 400)	*p*-Value *
**Socio-demographics**				
Age in years (mean ± SD)	48.9 (9.8)	47.4 (9.6)	50.2 (9.8)	0.0001
Education status (*N* (%))				<0.001
Illiterate	217 (28.8)	120 (34.0)	97 (24.2)	
Literate (informal)	100 (13.3)	59 (16.7)	41 (10.2)	
School	334 (44.4)	151 (42.7)	183 (45.7)	
College	102 (13.5)	23 (6.5)	79 (19.7)	
Religion (*N* (%))				0.054
Sikhism	423 (56.2)	214 (60.6)	209 (52.2)	
Hinduism	257 (34.1)	103 (29.2)	154 (38.5)	
Others	73 (9.7)	36 (10.2)	37 (9.2)	
**Medical/Family history**				
Diabetes (*N* (%))				0.001
Yes	75 (10.0)	21 (5.9)	54 (13.5)	
No	679 (90.0)	333 (94.1)	346 (86.5)	
Hypertension (*N* (%))				0.261
Yes	118 (15.6)	61 (17.2)	57 (14.2)	
No	636 (84.3)	293 (82.8)	343 (85.7)	
Family history of cancer (*N* (%))				0.009
Yes	129 (17.1)	47 (13.3)	82 (20.5)	
No	625 (82.9)	307 (86.7)	318 (79.5)	
**Anthropometrics**				
BMI (kg/m^2^) (mean ± SD)	27.1 (5.2)	27.0 (5.1)	27.2 (5.3)	0.5886
Waist circumference (cm) (mean ± SD)	94.5 (14.2)	93.0 (13.5)	95.9 (14.8)	0.0051
**Reproductive history**				
Age at menarche in years (mean ± SD)	14.8 (1.3)	14.9 (1.2)	14.7 (1.3)	0.0071
Age at first pregnancy in years (mean ± SD)	22.4 (3.4)	21.9 (2.8)	22.9 (3.7)	0.0002
Breastfeeding months/child (mean ± SD)	14.4 (12.2)	14.4 (13.4)	14.5 (11.1)	0.9547
Menopause (*N* (%))				0.033
No	308 (40.8)	159 (44.9)	149 (37.2)	
Yes	446 (59.1)	195 (55.0)	251 (62.7)	
Age at menopause in years (mean ± SD)	45.3 (6.1)	44.6 (6.1)	45.8 (6.1)	0.0357
Years of estrogen exposure (mean ± SD)	28.5 (6.7)	27.5 (6.7)	29.4 (6.6)	0.0001
Use of oral contraceptive pills (*N* (%))				0.873
Ever	48 (6.4)	22 (6.2)	26 (6.5)	
Never	706 (93.6)	332 (93.8)	374 (93.5)	
**Lifestyle factors**				
Physical Activity (median IQR)				
Moderate-vigorous (min/week)	180 (0, 515)	240 (0, 750)	140 (0, 420)	0.0003
Dietary Pattern ** (*N* (%))				<0.001
Lacto-vegetarian	155 (20.7)	56 (15.9)	99 (24.9)	
Lacto-ovo-vegetarian	466 (62.1)	255 (72.2)	211 (53.1)	
Non-vegetarian	129 (17.2)	42 (11.9)	87 (21.9)	

* Difference in mean/median/proportion between cases and controls by *t*-test/Wilcoxon Rank-Sum test/Chi-Square test, respectively. Missing data for different variables were <5%, and the maximum missing was for breastfeeding (4.6%) followed by BMI (4.3%). ** Duration of dietary pattern: 54% lifelong, 24% > 10 years, 2.4% 1–10 years, 8.1% < a year and missing 11.5%. SD: Standard Deviation; IQR: Inter Quartile Range.

**Table 2:** Associations between dietary patterns and breast cancer among study participants (*N* = 754 North Indian women aged 28 to 69 years).

Dietary Pattern	Age-Adjusted OR (95% CI) *N* = 684	*p*-Value	Fully-Adjusted * OR (95% CI) *N* = 640	*p*-Value
**With non-veg as reference**				
Non-vegetarian	Ref	-	Ref	-
Lacto-ovo-vegetarian	0.4 (0.3–0.7)	<0.001	0.6 (0.3–0.9)	0.033
Lacto-vegetarian	1.02 (0.6–1.7)	0.932	1.3 (0.7–2.4)	0.336
*p*-Interaction ** for menopausal status	0.1755		0.6158	
**With lacto-veg as reference**				
Lacto-vegetarian	Ref	-	Ref	-
Lacto-ovo-vegetarian	0.4 (0.3–0.6)	<0.001	0.4 (0.3–0.7)	<0.001
Non-vegetarian	0.97 (0.6–1.6)	0.932	0.7 (0.4–1.3)	0.336
*p*-Interaction ** for menopausal status	0.1007		0.3460	

* Unconditional logistic regression adjusted for age (years), state of residence (Punjab and Haryana), mode of recruitment (hospital- and population-based), education (categories), religion (categories), family history of cancer (Y/N), physical activity (mins/week), BMI (kg/m^2^), waist (cm), reproductive history (estrogen exposure (years), age at first pregnancy (years), breastfeeding per child (months)) and history of diabetes (Y/N) and hypertension (Y/N). Estrogen exposure: For postmenopausal women, calculated as the difference between age at menopause and age at menarche and for premenopausal women, calculated as the difference between current age and age at menarche. ** Likelihood-ratio test for interaction of menopausal status with dietary patterns (1. non-veg vs. lacto-ovo-veg; 2. lacto-veg vs. lacto-ovo-veg).

**Table 3:** Estimated daily consumption of macro- and micro-nutrients based on cases and controls in a sub-set of 298 study participants with FFQ data.

Nutrients Median (IQR)	Controls *N* = 128	Cases *N* = 170	*p*-Value *
Energy (kcal)	828.5 (593.4, 1234.5)	951.2 (558.8, 1183.6)	0.5982
Carbohydrates (g)	101.7 (72.1, 143.4)	102.4 (67.2, 145.6)	0.9659
Fat (g)	35.8 (25.1, 51.3)	41.3 (23.8, 54.0)	0.3001
Protein (g)	23.8 (16.5, 34.2)	27.7 (16.0, 34.6)	0.5407
Cholesterol (mg)	44.2 (18.5, 79.4)	56.4 (22.8, 144.0)	0.0250
Calcium (mg)	506.6 (288.7, 758.3)	614.7 (316.1, 815.0)	0.1417
Iron (mg)	7.0 (5.0, 9.2)	7.4 (4.9, 9.9)	0.8434
Zinc (mg)	3.4 (2.4, 4.8)	3.9 (2.2, 4.9)	0.5730
Alpha carotene (µg)	427.9 (243.4, 752.9)	521.7 (249.5, 726.4)	0.5965
Beta carotene (µg)	1749.7 (1121.4, 2952.7)	2166.3 (1051.2, 3169.1)	0.3007
Lycopene (µg)	394.6 (247.0, 610.2)	393.0 (265.9, 671.5)	0.4330
Luteine + Zeaxanthine (µg)	569.3 (421.2,871.7)	680.2 (384.5, 1079.0)	0.1825
Choline (mg)	56.5 (40.2, 81.0)	62.1 (37.4, 109.1)	0.3183
Vitamin C (mg)	51.3 (39.8, 72.1)	58.6 (37.5, 93.5)	0.1277
Folic Acid (mcg)	101.2 (68.1, 147.1)	103.5 (64.4, 147.2)	0.8572
Vitamin B12 (mcg)	1.1 (0.5, 2.1)	1.6 (0.7, 2.2)	0.1324

* Wilcoxon Rank-sum test for differences in median values between groups.

## Supplementary Materials

The following are available online at http://www.mdpi.com/1660-4601/15/9/1946/s1, Table S1: Characteristics of study participants (*N* = 750 North Indian women aged 28 to 69 years ^) based on their dietary patterns, Table S2: Associations between dietary patterns and breast cancer among a sub-set of study participants with FFQ data (*N* = 298 North Indian women aged 28 to 69 years), Table S3: Estimated daily consumption of macro- and micro-nutrients based on dietary pattern in a sub-set of 298 study participants with FFQ data, Table S4: Comparison of participant characteristics with (*N* = 298) and without nutrient data (*N* = 456).

## References

[1] Ferlay J., Soerjomataram I., Ervik M., Dikshit R., Eser S., Mathers C., Rebelo M., Parkin D.M., Forman D., Bray F. GLOBOCAN 2012: Cancer Incidence and Mortality Worldwide in 2012. http://globocan.iarc.fr.

[2] World Cancer Research Fund Internationa (2014). Diet, Nutrition, Physical Activity and Breast Cancer Survivors.

[3] Keum N., Lee D.H., Marchand N., Oh H., Liu H., Aune D., Greenwood D.C., Giovannucci E.L. (2015). Egg intake and cancers of the breast, ovary and prostate: A dose-response meta-analysis of prospective observational studies. Br. J. Nutr..

[4] Appleby P.N., Key T.J. (2016). The long-term health of vegetarians and vegans. Proc. Nutr. Soc..

[5] Dandamudi A., Tommie J., Nommsen-Rivers L., Couch S. (2018). Dietary patterns and breast cancer risk: A systematic review. Anticancer Res..

[6] Global Burden of Disease Study 2016 (GBD 2016) Results. http://ghdx.healthdata.org/gbd-results-tool.

[7] Thakur J., Budukh A., Kapoor R., Malhotra P., Bashar A., Kathirvel S., Dixit R., Arora P., Bhatia S., Badwe R. (2017). Urban–rural differences in cancer incidence and pattern in Punjab and Chandigarh: Findings from four new population-based cancer registries in North India. Int. J. Noncommun. Dis..

[8] National Centre for Disease Informatics and Research, NCRP, Indian Council of Medical Research (2016). Three-Year Report of Population Based Cancer Registries 2012–2014.

[9] Malvia S., Bagadi S.A., Dubey U.S., Saxena S. (2017). Epidemiology of breast cancer in Indian women. Asia Pac. J. Clin. Oncol..

[10] Kakarala M., Rozek L., Cote M., Liyanage S., Brenner D.E. (2010). Breast cancer histology and receptor status characterization in Asian Indian and Pakistani women in the U.S.—A SEER analysis. BMC Cancer.

[11] Leong S.P., Shen Z.Z., Liu T.J., Agarwal G., Tajima T., Paik N.S., Sandelin K., Derossis A., Cody H., Foulkes W.D. (2010). Is breast cancer the same disease in Asian and Western countries?. World J. Surg..

[12] National Family Health Survey I. http://www.rchiips.org/nfhs/.

[13] Sinha R., Anderson D.E., McDonald S.S., Greenwald P. (2003). Cancer risk and diet in India. J. Postgrad. Med..

[14] Shridhar K., Dhillon P.K., Bowen L., Kinra S., Bharathi A.V., Prabhakaran D., Reddy K.S., Ebrahim S., Indian Migration Study Group (2014). The association between a vegetarian diet and cardiovascular disease (CVD) risk factors in India: The Indian migration study. PLoS ONE.

[15] Shridhar K., Dhillon P.K., Bowen L., Kinra S., Bharathi A.V., Prabhakaran D., Reddy K.S., Ebrahim S. (2014). Nutritional profile of Indian vegetarian diets—The Indian Migration Study (IMS). Nutr. J..

[16] Green R., Milner J., Joy E.J., Agrawal S., Dangour A.D. (2016). Dietary patterns in India: A systematic review. Br. J. Nutr..

[17] Shridhar K., Satija A., Dhillon P.K., Agrawal S., Gupta R., Bowen L., Kinra S., Bharathi A.V., Prabhakaran D., Srinath Reddy K. (2018). Association between empirically derived dietary patterns with blood lipids, fasting blood glucose and blood pressure in adults—The India migration study. Nutr. J..

[18] Arnold F., Parasuraman S., Arokiasamy P., Kothari M., International Institute for Population Sciences (2009). Nutrition in India National Family Health Survey, I. NFHS 3.

[19] Rao D.N., Ganesh B., Desai P.B. (1994). Role of reproductive factors in breast cancer in a low-risk area: A case-control study. Br. J. Cancer.

[20] Jayalekshmi P., Varughese S.C., Kalavathi Nair M.K., Jayaprakash V., Gangadharan P., Nair R.R., Akiba S. (2009). A nested case-control study of female breast cancer in Karunagappally cohort in Kerala, India. Asian Pac. J. Cancer Prev..

[21] Kamath R., Mahajan K.S., Ashok L., Sanal T.S. (2013). A study on risk factors of breast cancer among patients attending the tertiary care hospital, in Udupi District. Indian J. Commun. Med..

[22] Balasubramaniam S.M., Rotti S.B., Vivekanandam S. (2013). Risk factors of female breast carcinoma: A case control study at Puducherry. Indian J. Cancer.

[23] Dos Santos Silva I., Mangtani P., McCormack V., Bhakta D., Sevak L., McMichael A.J. (2002). Lifelong vegetarianism and risk of breast cancer: A population-based case-control study among South Asian migrant women living in England. Int. J. Cancer.

[24] Kamath S.K., Murillo G., Chatterton R.T., Hussain E.A., Amin D., Mortillaro E., Peterson C.T., Alekel D.L. (1999). Breast cancer risk factors in two distinct ethnic groups: Indian and Pakistani vs. American premenopausal women. Nutr. Cancer.

[25] Gathani T., Barnes I., Ali R., Arumugham R., Chacko R., Digumarti R., Jivarajani P., Kannan R., Loknatha D., Malhotra H. (2017). Lifelong vegetarianism and breast cancer risk: A large multicentre case control study in India. BMC Womens Health.

[26] Bowen L., Bharathi A.V., Kinra S., Destavola B., Ness A., Ebrahim S. (2012). Development and evaluation of a semi-quantitative food frequency questionnaire for use in urban and rural India. Asia Pac. J. Clin. Nutr..

[27] Ravindran R.D., Vashist P., Gupta S.K., Young I.S., Maraini G., Camparini M., Jayanthi R., John N., Fitzpatrick K.E., Chakravarthy U. (2011). Inverse association of vitamin C with cataract in older people in India. Ophthalmology.

[28] Ravindran R.D., Vashist P., Gupta S.K., Young I.S., Maraini G., Camparini M., Jayanthi R., John N., Fitzpatrick K.E., Chakravarthy U. (2011). Prevalence and risk factors for vitamin C deficiency in north and south India: A two centre population based study in people aged 60 years and over. PLoS ONE.

[29] Department of Chronic Diseases and Health Promotion WHO Global Physical Activity Questionaire 2. http://www.who.int/chp/steps/GPAQ%20Instrument%20and%20Analysis%20Guide%20v2.pdf.

[30] Godos J., Bella F., Sciacca S., Galvano F., Grosso G. (2017). Vegetarianism and breast, colorectal and prostate cancer risk: An overview and meta-analysis of cohort studies. J. Hum. Nutr. Diet..

[31] Agrawal S., Millett C.J., Dhillon P.K., Subramanian S.V., Ebrahim S. (2013). Type of vegetarian diet, obesity and diabetes in adult Indian population. Nutr. J..

[32] Missmer S.A., Smith-Warner S.A., Spiegelman D., Yaun S.S., Adami H.O., Beeson W.L., van den Brandt P.A., Fraser G.E., Freudenheim J.L., Goldbohm R.A. (2002). Meat and dairy food consumption and breast cancer: A pooled analysis of cohort studies. Int. J. Epidemiol..

[33] Si R., Qu K., Jiang Z., Yang X., Gao P. (2014). Egg consumption and breast cancer risk: A meta-analysis. Breast Cancer.

[34] Wirfalt E., Li C., Manjer J., Ericson U., Sonestedt E., Borgquist S., Landberg G., Olsson H., Gullberg B. (2011). Food sources of fat and sex hormone receptor status of invasive breast tumors in women of the Malmo Diet and Cancer cohort. Nutr. Cancer.

[35] Goodman M., Bostick R.M., Kucuk O., Jones D.P. (2011). Clinical trials of antioxidants as cancer prevention agents: Past, present, and future. Free Radic. Biol. Med..

