# Supplementary material for: Dietary Patterns and Breast Cancer Risk: A Multi-Centre Case Control Study among North Indian Women
Source: Int J Environ Res Public Health. 2018 Sep 6;15(9):1946. doi: 10.3390/ijerph15091946 (PMC6164652; doi:10.3390/ijerph15091946)
Supplement: Supplementary file 1 [file ijerph-15-01946-s001.pdf]

International Journal of
*Environmental Research and Public Health*

MDPI

## Supplementary Materials

**Supplementary Table S1.** Characteristics of study participants (*N* = 750 North Indian women aged 28 to 69 years ^) based on their dietary patterns.

| Characteristics | Lacto-veg *N* = 155 | Ovo-veg *N* = 466 | Non-veg *N* = 129 | *p*-Value |
|---|---|---|---|---|
| Age (years), mean (SD) | 47.9 (9.6) | 48.8 (10.0) | 50.7 (9.5) | 0.048 |
| Education *N* (%) | | | | |
| Illiterate | 54 (34.8) | 137 (29.5) | 26 (20.2) | |
| No formal education | 14 (9.0) | 79 (17.0) | 7 (5.4) | <0.001 |
| School | 68 (43.9) | 194 (41.7) | 70 (54.3) | |
| College | 19 (12.3) | 55 (11.8) | 26 (20.2) | |
| Religion (*N* (%)) | | | | |
| Sikhism | 89 (57.4) | 276 (59.3) | 57 (44.2) | <0.001 |
| Hinduism | 46 (29.7) | 140 (30.1) | 68 (52.7) | |
| Others | 20 (12.9) | 49 (10.5) | 4 (3.1) | |
| Family history of cancer (*N* (%)) | | | | |
| Yes | 15 (9.7) | 85 (18.2) | 29 (22.5) | 0.011 |
| No | 140 (90.3) | 381 (81.8) | 100 (77.5) | |
| Diabetes | | | | |
| No | 141 (91.0) | 429 (92.1) | 105 (81.4) | 0.002 |
| Yes | 14 (9.0) | 37 (7.9) | 24 (18.6) | |
| Hypertension | | | | |
| No | 139 (89.7) | 399 (85.6) | 94 (72.9) | <0.001 |
| Yes | 16 (10.3) | 67 (14.4) | 35 (27.1) | |
| BMI (Kg/m2), mean (SD) | 26.8 (5.6) | 26.8 (5.0) | 28.3 (5.4) | 0.012 |
| Waist circumference, mean (SD) | 96.1 (16.1) | 92.6 (14.1) | 99.5 (10.9) | <0.001 |
| Age at menarche (years) mean (SD) | 14.7 (1.3) | 14.8 (1.3) | 14.8 (1.4) | 0.37 |
| Menopause (*N* (%)) | | | | |
| Yes | 93 (60.0) | 265 (56.9) | 86 (66.7) | 0.13 |
| No | 62 (40.0) | 201 (43.1) | 43 (33.3) | |
| Age at menopause in years (mean ± SD) | 45.6 (6.0) | 45.1 (6.2) | 45.6 (5.9) | 0.7584 |
| Years of estrogen exposure (mean ± SD) | 28.8 (6.9) | 28.2 (6.6) | 29.4 (6.9) | 0.21 |
| Ever Pregnant (*N* (%)) | | | | |
| No | 5 (3.2) | 8 (1.7) | 4 (3.1) | 0.43 |
| Yes | 150 (96.8) | 458 (98.3) | 125 (96.9) | |
| Age at first pregnancy (years), mean (SD) | 21.7 (4.0) | 22.4 (3.1) | 23.3 (3.3) | <0.001 |
| Total pregnancies (*N* (%)) | | | | |
| 1 | 6 (4.0) | 37 (8.1) | 2 (1.6) | 0.028 |
| 2 | 28 (18.7) | 107 (23.4) | 31 (23.8) | |
| 3+ | 116 (77.3) | 314 (68.6) | 92 (73.6) | |
| Total breastfeeding months/child, median (IQR) | 13.8 (11.8) | 13.8 (12.7) | 17.3 (10.7) | 0.0156 |
| Physical activity, median (IQR) | | | | |
| Moderate-vigorous (min/week) | 240 (0, 1080) | 180 (0, 525) | 160 (0, 420) | 0.0027 |

* Difference in mean/median/proportion among the groups by ANOVA, Kruskal-Wallis ANOVA/ Chi-Square test, respectively. ^ Information on dietary pattern missing for 4 participants.

**Supplementary Table S2.** Associations between dietary patterns and breast cancer among a sub-set of study participants with FFQ data (*N* = 298 North Indian women aged 28 to 69 years).

| Dietary Pattern | Age-Adjusted OR (95% CI) *N* = 258 | *p*-Value | Fully-adjusted * OR (95% CI) *N* = 247 | *p*-Value |
|---|---|---|---|---|
| With non-veg as reference | | | | |

| | | | | |
|---|---|---|---|---|
| Non-vegetarian | Ref | - | Ref | - |
| Lacto-ovo-vegetarian | 0.2 (0.1–0.4) | <0.001 | 0.2 (0.1–0.5) | 0.001 |
| Lacto-vegetarian | 0.4 (0.1–1.1) | 0.054 | 0.5 (0.1–1.6) | 0.234 |
| p-Interaction ** for menopausal status | 0.4368 | | 0.3204 | |
| **With lacto-veg as reference** | | | | |
| Lacto-vegetarian | Ref | - | Ref | - |
| Lacto-ovo-vegetarian | 0.4 (0.2–0.9) | 0.027 | 0.4 (0.1–0.9) | 0.032 |
| Non-vegetarian | 2.6 (0.98–6.8) | 0.054 | 1.2 (0.6–6.4) | 0.234 |
| *p*-Interaction ** for menopausal status | 0.2237 | | 0.0712 | |

* Dnconditional logistic regression adjusted for age (years), state of residence (Punjab and Haryana), mode of recruitment (hospital- and population-based), education (categories), religion (categories), family history of cancer (Y/N), physical activity (mins/week), BMI (kg/m2), waist (cm), reproductive history (estrogen exposure (years), age at first pregnancy (years), breastfeeding per child (months)) and history of diabetes (Y/N) and hypertension (Y/N). Estrogen exposure: For postmenopausal women, calculated as the difference between age at menopause and age at menarche and for premenopausal women, calculated as the difference between current age and age at menarche. ** Likelihood-ratio test for interaction of menopausal status with dietary patterns (1. non-veg vs. lacto-ovo-veg; 2. lacto-veg vs. lacto-ovo-veg).

**Supplementary Table 3.** Estimated daily consumption of macro- and micro-nutrients based on dietary pattern in a sub-set of 298 study participants with FFQ data.

| Nutrients Median (IQR) | Total ^ $N$ = 298 | Lacto-vegetarian $N$ = 48 | Ovo-vegetarian $N$ = 188 | Non-vegetarian $N$ = 60 | $p$-Value * |
|---|---|---|---|---|---|
| Energy (kcal) | 890.9 (569.2, 1198.1) | 934.9 (653.2, 1146.8) | 882.7 (562.9, 1254.2) | 890.3 (564.8, 1123.7) | 0.76 |
| Carbohydrates (g) | 101.7 (69.0, 145.2) | 103.0 (69.0, 144.4) | 102.3 (71.9, 150.3) | 98.9 (61.4, 123.3) | 0.16 |
| Fat (g) | 39.6 (24.8, 53.8) | 37.4 (25.1, 50.8) | 38.7 (24.9, 53.7) | 42.4 (22.9, 58.5) | 0.55 |
| Protein (g) | 25.6 (16.2, 34.4) | 27.4 (16.8, 33.9) | 25.3 (16.8, 35.3) | 25.9 (15.5, 32.2) | 0.63 |
| Cholesterol (mg) | 51.0 (20.8,115.4) | 44.4 (18.1, 107.2) | 40.6 (17.1, 63.0) | 152.7 (95.5, 295.3) | 0.0001 |
| Calcium (mg) | 549.7 (295.4, 790.0) | 479.1 (277.2, 748.2) | 534.0 (296.1, 766.8) | 656.6 (335.3, 913.3 | 0.19 |
| Iron (mg) | 7.3 (5.0, 9.8) | 7.4 (5.5, 9.6) | 7.3 (5.0, 10.2) | 7.1 (4.0, 9.4) | 0.30 |
| Zinc (mg) | 3.6 (2.3, 4.9) | 3.5 (2.1, 4.7) | 3.6 (2.3, 5.0) | 3.6 (2.3, 4.6) | 0.57 |
| Choline (mg) | 59.5 (38.1, 99.8) | 59.2 (39.7, 95.4) | 56.5 (37.9, 89.0) | 85.9 (40.1, 138.2) | 0.0392 |
| Alpha carotene (µg) | 477.8 (246.7, 741.7) | 547.5 (283.9, 849.7) | 409.2 (242.9, 671.7) | 533.1 (247.6, 913.0) | 0.2100 |
| Beta carotene (µg) | 1896.5 (1091.1, 3122.1) | 1993.1 (1212.1, 3100.9) | 1748.3 (1000.6, 2739.3) | 2701.7 (1307.1, 4088.5) | 0.0071 |
| Lycopene (µg) | 394.0 (258.9, 638.9) | 438.5 (287.4, 655.7) | 373.2 (247.2, 555.9) | 551.3 (283.3, 700.1) | 0.1242 |
| Luteine+Zeaxanthine (µg) | 608.9 (404.4, 991.2) | 680.4 (412.3, 971.6) | 565.9 (387.1, 914.6) | 829.7 (515.6, 1260.2) | 0.0200 |
| Vitamin C (mg) | 54.5 (38.8, 84.5) | 51.3 (36.1, 67.4) | 51.4 (38.2, 82.5) | 71.3 (44.0, 98.0) | 0.058 |
| Folic Acid (µg) | 102.1 (67.8, 147.1) | 105.9 (68.3, 156.4) | 102.6 (70.2, 148.5) | 94.8 (58.8, 128.7) | 0.33 |
| Vitamin B12 (µg) | 1.5 (0.6, 2.2) | 1.1 (0.4, 2.2) | 1.3 (0.6, 2.1) | 2.0 (1.0, 2.7) | 0.006 |

* Kruskal-Wallis rank test for differences in median values among groups; ^ Information on dietary pattern missing for 2 participants.

**Supplementary Table S4.** Comparison of participant characteristics with (*N* = 298) and without nutrient data (*N* = 456).

| Baseline Characteristics | Participants with Nutrient Data | Participants Without Nutrient Data | *p*-Value * |
|---|---|---|---|
| ***N*** | **298** | **456** | |
| Age (years), median (IQR) | 50.0 (42.0, 58.0) | 48.0 (41.0, 56.0) | 0.091 |
| Education *N* (%) | | | |
| Illiterate | 96 (32.3%) | 121 (26.5%) | |
| No formal education | 51 (17.2%) | 49 (10.7%) | 0.001 |
| School | 123 (41.4%) | 211 (46.3%) | |
| College | 27 (9.1%) | 75 (16.4%) | |
| Diabetes *N* (%) | | | |
| No | 265 (88.9%) | 425 (91.0%) | 0.35 |
| Yes | 33 (11.1%) | 42 (9.0%) | |
| Hypertension *N* (%) | | | |
| No | 263 (88.3%) | 384 (82.2%) | 0.024 |
| Yes | 35 (11.7%) | 83 (17.8%) | |
| BMI (Kg/m2), mean (SD) | 26.5 (5.3) | 27.5 (5.1) | 0.016 |
| Waist circumference, mean (SD) | 92.8 (14.1) | 95.7 (14.2) | 0.009 |
| Years of estrogen exposure (mean ± SD) | 28.4 (6.8) | 28.6 (6.6) | 0.6870 |
| Breastfeeding months /child (mean ± SD) | 12.0 (12.0) | 16.0 (12.1) | <0.001 |
| Age at first pregnancy in years (mean ± SD) | 22.3 (3.0) | 22.5 (3.6) | 0.3642 |
| Physical activity, median (IQR) Moderate-vigorous (min/week) | 210 (0, 757.5) | 180 (0,420) | 0.1262 |
| Tobacco use *N* (%) | | | |
| No | 297 (99.7%) | 453 (98.7%) | 0.17 |
| Yes | 1 (0.3%) | 6 (1.3%) | |

* *p*-Values for differences in proportion, mean or median values between groups are from Chi-square test, *t*-test or Wilcoxon Rank-sum test.
